# Development and validation of nomogram prognostic model for early-stage T1-2N0M0 small cell lung cancer: A population-based analysis

**DOI:** 10.3389/fonc.2022.921365

**Published:** 2022-11-17

**Authors:** Tao Ge, Shuncang Zhu, Liangdong Sun, Laibo Yin, Jie Dai, Jiayi Qian, Xiangru Chen, Peng Zhang, Jialong Zhu, Gening Jiang

**Affiliations:** ^1^ Department of Thoracic Surgery, Shanghai Pulmonary Hospital, Tongji University School of Medicine, Shanghai, China; ^2^ Department of Thoracic and Cardiovascular Surgery, The First Affiliated Hospital, School of Medicine, Shihezi University, Shihezi, China

**Keywords:** small cell lung cancer, nomogram, prognosis, Surveillance, Epidemiology, and End Results (SEER), lung cancer-specific survival

## Abstract

**Background:**

Survival outcomes of early-stage T1-2N0M0 small cell lung cancer (SCLC) patients differ widely, and the existing Veterans Administration Lung Study Group (VALSG) or TNM staging system is inefficient at predicting individual prognoses. In our study, we developed and validated nomograms for individually predicting overall survival (OS) and lung cancer-specific survival (LCSS) in this special subset of patients.

**Methods:**

Data on patients diagnosed with T1-2N0M0 SCLC between 2000 and 2015 were extracted from the Surveillance, Epidemiology, and End Results (SEER) database. All enrolled patients were split into a training cohort and a validation cohort according to the year of diagnosis. Using multivariable Cox regression, significant prognostic factors were identified and integrated to develop nomograms for 1-, 3-, and 5-year OS and LCSS prediction. The prognostic performance of our new model was measured by the concordance index (C-index) and calibration curve. We compared our latest model and the 8th AJCC staging system using decision curve analyses (DCA). Kaplan–Meier survival analyses were applied to test the application of the risk stratification system.

**Results:**

A total of 1,147 patients diagnosed from 2000 to 2011 were assigned to the training cohort, and 498 cases that were diagnosed from 2012 to 2015 comprised the validation cohort. Age, surgery, lymph node removal (LNR), and chemotherapy were independent predictors of LCSS. The variables of sex, age, surgery, LNR, and chemotherapy were identified as independent predictors of OS. The above-mentioned prognostic factors were entered into the nomogram construction of OS and LCSS. The C-index of this model in the training cohort was 0.663, 0.702, 0.733, and 0.658, 0.702, 0.733 for predicting 1-, 3-, and 5-year OS and LCSS, respectively. Additionally, in the validation cohort, there were 0.706, 0.707, 0.718 and 0.712, 0.691, 0.692. The calibration curve showed accepted prediction accuracy between nomogram-predicted survival and actual observed survival, regardless of OS or LCSS. In addition, there were significant distinctions in the survival curves of OS and LCSS between different risk groups stratified by prognostic scores. Compared with the 8th AJCC staging system, our new model also improved net benefits.

**Conclusions:**

We developed and validated novel nomograms for individual prediction of OS and LCSS, integrating the characteristics of patients and tumors. The model showed superior reliability and may help clinicians make treatment strategies and survival predictions for early-stage T1-2N0M0 SCLC patients.

## Introduction

Lung cancer remains the leading cause of cancer-related deaths and an important public health concern affecting both men and women worldwide (1). Small cell lung cancer (SCLC) accounts for approximately 15%–20% of lung cancer patients and is characterized by high vascularity, high cellular proliferation, rapid progression, and early metastatic spread (2). Approximately 30% of SCLC patients were non-metastatic at initial diagnosis (3). Compared to NSCLC, for which the 5-year survival rate is 18%, SCLC has only a 6.2% 5-year survival rate (4).

With the improvement of patients’ health awareness and the popularity of computed tomography (CT) screening for long-term smokers, it is more likely to increase the incidence of early-stage lung cancer diagnosis (5). However, approximately 5% of SCLC patients present with early-stage T1-2N0M0 disease, which has a better prognosis with a 5-year survival rate of up to 50% (6–8). In this group of patients, a surgical approach can be proposed as part of multidisciplinary treatment after excluding mediastinal lymph node involvement, according to the current National Comprehensive Cancer Network (NCCN) guidelines (9). Most previous clinical guidelines and treatment strategies for SCLC were based on the Veterans Administration Lung Study Group (VALSG) staging system, in which SCLC patients were roughly divided into limited-stage and extensive-stage. However, individual survival differs widely at the same stage. It has been recommended that the American Joint Committee on Cancer (AJCC) TNM staging system replace the VALSG staging system because the TNM system could contribute to more appropriate treatment selections and more precise assessments of prognosis (10, 11). Nevertheless, in addition to the TNM staging status, some studies have revealed that clinical characteristics like sex, age, location, surgical procedure, adjuvant chemotherapy, or radiotherapy were also noteworthy factors influencing individual survival outcomes of SCLC cancer patients (12–14). Hence, a more refined model with better individualized prognostic discrimination is required to solve this problem.

Nomogram models have been widely recognized as a feasible tool to predict individual prognosis for cancer patients and could benefit treatment strategy-making and clinical trials (15, 16). It mainly depends on both patient and disease features. The unique advantage of the nomogram is that it provides the score of each influencing factor according to the contribution degree of each influencing factor in the regression model. It then calculates the total score of an individual to obtain the predicted value of the individual. To date, there have been no nomogram studies regarding the OS and LCSS of early-stage T1-2N0M0 SCLC patients. Thus, the objective of this study was to derive and validate a prognostic nomogram to quantitatively predict survival outcomes in early-stage T1-2N0M0 SCLC patients using a large cohort from the SEER database, which would help clinicians make better clinical decisions and further improve the survival of patients.

## Methods

### Patient selection

The SEER is an authoritative source for cancer statistics that covers approximately 28% of the US population and contains data on cancer occurrences in 18 areas of the United States, which can help reduce the cancer burden among the U.S. population. The data of 100,585 patients diagnosed with SCLC from 2000 to 2015 were retrieved from the SEER database using SEER*Stat version 8.3.9 (National Cancer Institute, Bethesda, MD, USA). The study cohort consisted of patients with the following International Classification of Disease for Oncology, Third Edition (ICD-O-3), morphology codes: 8041/3, 8042/3, 8043/3, 8044/3, and 8045/3; and the site codes: C34.0, C34.1, C34.2, C34.3, C34.8, and C34.9. The exclusion criteria were as follows: (I) not receiving regular follow-up or no follow-up; (II) patients having at least one prior malignancy; (III) not being pathologically confirmed by immunohistochemistry; and (IV) patients with missing information concerning the primary tumor size (T), regional lymph node (N), or distant metastasis (M) stage and clinical information. After that, we also excluded patients who underwent pneumonectomy and whose information about the removed lymph nodes was unknown.

### Variables

The extracted clinical information contained sex, age (<65 years, ≥65 years), race, primary site (upper lobe, lower lobe, middle lobe, main bronchus, overlapping lesion), laterality (left, right), tumor size (≤3 cm, 3–4 cm, 4–5 cm), pathological grade (moderate, well, poor, undifferentiated, unknown), surgery (lobectomy, others, non-surgery), lymph nodes removal (LNR) (<4, ≥4 regional lymph nodes), radiotherapy or not, chemotherapy or not, survival months, causes of death, vital status. In terms of surgery, all other surgical approaches are classified as others except for lobectomy because the number of some surgical procedures is so limited that we cannot analyze them separately. In addition, we converted the TNM stages based on the 6/7th edition to those of the 8th AJCC staging system for each patient using tumor size, tumor CS extension, and the 6/7th edition N/M stages (17). We assembled the tumor sizes of ≤1 cm, 1–2 cm, and 2–3 cm as the group of ≤3 cm because no significant difference in survival was found among these patients (18). For chemotherapy or radiotherapy, we were unable to define neoadjuvant or adjuvant therapy due to the lack of sequence of the treatment. Regretfully, the information on the visceral pleural invasion for lung cancer was unavailable before 2010. The information regarding prophylactic cranial radiation was missing during this period. The primary outcomes were defined as OS and LCSS. The time of the last follow-up was November 2020. OS was defined as the interval between cancer diagnosis and death resulting from any cause or the last follow-up for patients still alive. LCSS was defined as the length of time from cancer diagnosis to death from lung cancer.

### Development and evaluation of the nomogram prognostic model

According to our exclusion criteria, 1,645 patients were included for analysis. A total of 1,147 patients who were diagnosed from 2000 to 2011 were assigned to the training cohort and used to develop the nomogram prognostic models. Four hundred and ninety-eight patients diagnosed from 2012 to 2015 were assigned to the validation cohort and used to validate the model. To identify independent prognostic factors for OS and LCSS to build our prognosis model, we performed a univariate COX proportional hazard regression analysis. Significant factors from the univariate analysis (P-value <0.05) entered the multivariate COX proportional hazard regression analysis to obtain the hazard ratio (HR) and corresponding 95% confidential interval (CI) for every independent prognostic variable. The prognostic nomograms for OS and LCSS were constructed based on the risk factors calculated by the final multivariate Cox regression model.

The performance and evaluation of a nomogram mainly depend on two facets: discrimination and calibration accuracy. The discrimination refers to the efficiency of the model to distinguish patients with different survival outcomes. The concordance index (C-index) is recognized as a tool to measure discrimination and represents a concordance measure analogous to the area under the receiver operating characteristic (ROC). The theoretical value of the C-index ranges from 0 (indicating no better than random chance) to 1.0 (indicating perfect prediction) (16). Calibration curves of the nomogram for 1-, 3-, and 5-year survival were plotted to measure the consistency between predicted survival probability and actual survival proportion in the training and validation cohorts. A perfectly calibrated model would present a 45-degree curve. The estimation of discrimination and calibration was performed by bootstrapping 1,000 times. The conventional staging model, the AJCC 8th TNM staging system, was also assessed for prognostic performance. In addition, the area under the curve (AUC) of the time-dependent ROC was calculated for each month, from months 1 to 60. We conducted the comparisons of AUCs between the proposed nomogram and the AJCC 8th staging system. The decision curve analysis (DCA) was also conducted to evaluate the benefits and advantages of our new predicting model over the existing 8th edition AJCC TNM staging system (19).

The patients of every cohort were divided into two different risk groups (low-risk and high-risk) according to the prognostic scores of every patient on the nomogram. The cut-off values were defined using the X-tile software version 3.6.1 (Copyright: Camp/Rimm, Yale University), which could recognize the optimal cut-off values for continuous variables by calculating the largest Chi-square and minimum *p*-values. These cut-off values were then applied to the different TNM categories and the validation cohort; the respective log-rank P values were calculated to compare the difference in survival.

### Statistical analysis

Chi-squared tests were used to analyze the categorical variables. OS and LCSS survival analyses were performed using the Kaplan–Meier method. Kaplan–Meier survival analysis was used to assess distinctions in prognosis with a log-rank P-value. A two-sided P-value <0.05 was deemed significant. All data analyses were performed using SPSS 26.0 (SPSS, Chicago, IL) and RStudio version 4.1.0 (RStudio, Boston, MA, USA). The R packages ‘survival,’ ‘rms,’ ‘riskRegression,’ ‘survminer,’ and ‘ggDCA’ were used for nomogram construction and evaluation. Furthermore, the R packages ‘DynNom,’ ‘DNbuilder,’ and ‘rsconnect’ were applied for developing a user-friendly web-based interface for our nomogram.

## Results

### Characteristics of the training and validation cohorts

**Figure 1:**
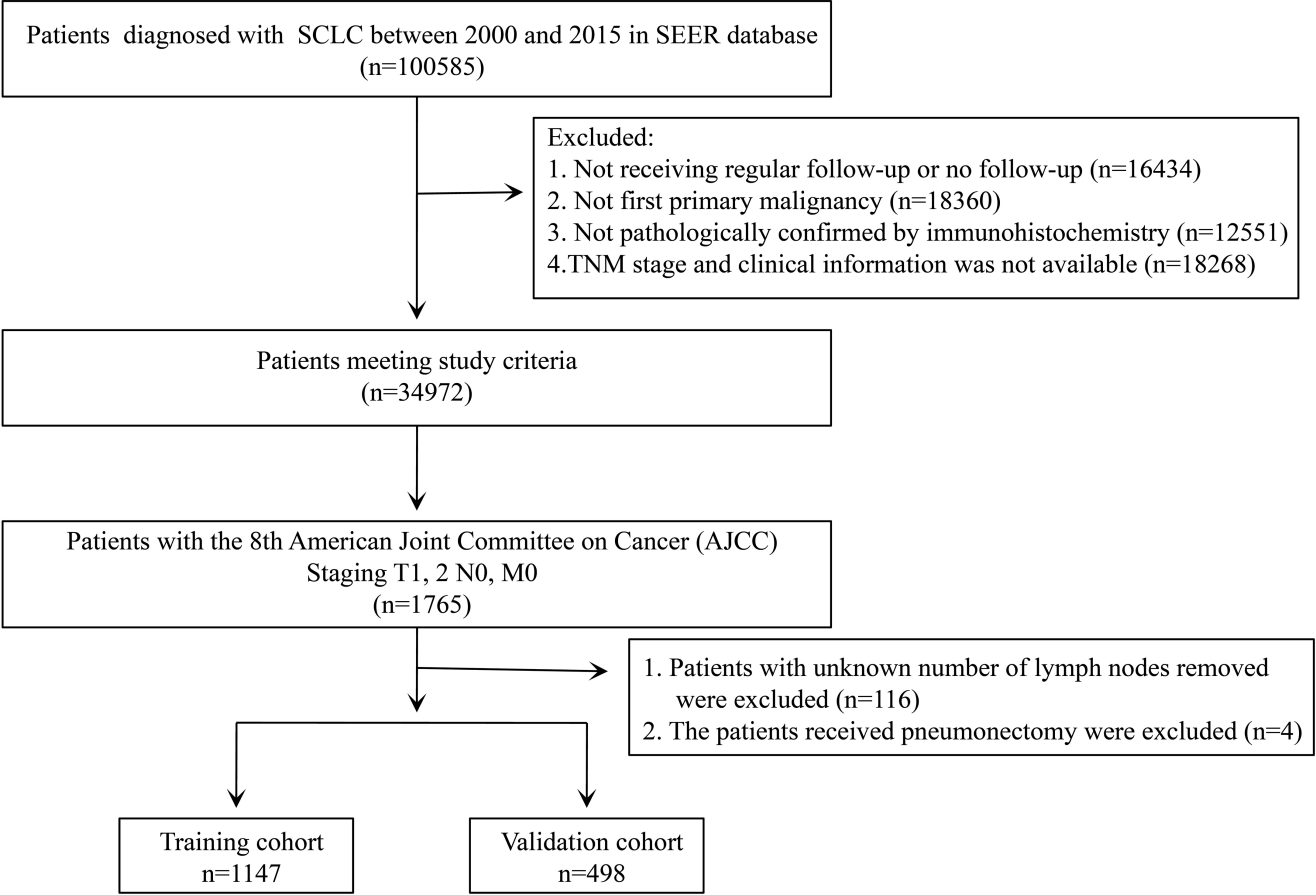
The flow chart shows the process of patient selection.

We selected 1,645 eligible patients with stage T1-2N0M0 (**Figure 1**). The distribution of clinical characteristics of patients and pathological characteristics of tumors is presented in **Table 1**. Based on the year of diagnosis, the included patients were divided into two distinct cohorts: 1,147 patients diagnosed from 2000 to 2011 were assigned to the training cohort, whereas cases that were diagnosed from 2012 to 2015 were used as the validation cohort (n = 498). In the training cohort, females were the predominant sex. Most of the patients were aged ≥65 years old. Caucasians were the predominant race. The most common site of the tumor was the upper lobe, followed by the lower lobe. The number of patients with right-side primary tumors was 653 (56.93%) in the training cohort. The distribution of tumor size was 61.38%, 22.14%, and 16.48% for ≤3 cm, 3–4 cm, and 4–5 cm, respectively. Over half of the patients did not receive surgery. Lobectomy was the predominant surgical approach in the patients receiving surgical treatment, and most patients underwent lymph node removal of fewer than four lymph nodes. In the training cohort, more than half of the patients were treated with chemotherapy and radiotherapy. The above demographics of the patients in the validation cohort were similar to those in the training cohort. The differentiated grade of most tumors in both cohorts was unknown. In comparing the training and validation cohorts, the demographic variables were not significant, except for the variables of grade and LNR.

**Table 1:** Demographic and clinicopathologic characteristics of the training and validation cohort.

Characteristics	Training cohort (%)	Validation cohort (%)	P-value
No. of cases	1,147	498	
Sex			0.056
Male	544 (47.43)	210 (42.17)	
Female	603 (52.57)	288 (57.83)	
Age			0.206
<65 y	367 (32.00)	143 (28.71)	
≥65 y	780 (68.00)	355 (71.29)	
Race			0.097
White	1,020 (88.93)	424 (85.14)	
Black	89 (7.76)	51 (10.24)	
Others	38 (3.31)	23 (4.62)	
Primary site			0.522
Upper lobe	664 (57.89)	304 (61.04)	
Lower lobe	341 (29.73)	145 (29.12)	
Middle lobe	75 (6.54)	27 (5.42)	
Main bronchus	64 (5.58)	20 (4.02)	
Overlapping lesion	3 (0.26)	2 (0.40)	
Laterality			1.000
Left	494 (43.07)	215 (43.17)	
Right	653 (56.93)	283 (56.83)	
Tumor size (cm)			0.050
≤3	704 (61.38)	333 (66.87)	
3–4	254 (22.14)	104 (20.88)	
4–5	189 (16.48)	61 (12.25)	
Grade			<0.001
Moderate + well	18 (1.57)	11 (2.21)	
Poor	200 (17.44)	95 (19.08)	
Undifferentiated	368 (32.08)	86 (17.27)	
Unknown	561 (48.91)	306 (61.45)	
Surgery			0.104
Non-surgery	825 (71.93)	346 (69.48)	
Lobectomy	181 (15.78)	99 (19.88)	
Others	141 (12.29)	53 (10.64)	
LNR(number)			0.005
<4	976 (85.09)	395 (79.32)	
≥4	171 (14.91)	103 (20.68)	
Chemotherapy			0.593
No	363 (31.65)	165 (33.13)	
Yes	784 (68.35)	333 (66.87)	
Radiotherapy			0.373
No	568 (49.52)	234 (46.99)	
Yes	579 (50.48)	264 (53.01)	

LNR, lymph node removal.

### Independent prognostic factors in the training cohort

There were 1,027 events (deaths) in the training cohort, of which 799 patients died of cancer. The mean follow-up duration was 38.30 months (median, 20 months; range, 1–225 months). In univariate analysis, sex, age, tumor size, surgery, LNR, and chemotherapy were significantly associated with OS (**Table 2**), and these factors were also significantly associated with LCSS except for the factor of sex (**Table 3**). After multivariate analysis, sex, age, surgery, LNR, and chemotherapy were proven to be associated with OS (**Table 2**). In the group aged ≥65 years, no surgery, less than four regional lymph nodes removed, and no chemotherapy were demonstrated to have a higher hazard of death from lung cancer through multivariate analysis (**Table 3**). In terms of surgical procedures, lobectomy was associated with the lowest risk of death.

**Table 2:** Univariable and multivariate Cox proportional hazards regression analysis for overall survival.

Variables	Univariate cox regression		Multivariate cox regression	
	HR (95% CI)	P-value	HR (95% CI)	P-value
Sex				
Female	1		1	
Male	1.23 (1.08–1.39)	0.001	1.13 (1.00–1.28)	0.045
Age				
<65 y	1		1	
≥65 y	1.53 (1.34–1.76)	<0.001	1.49 (1.29–1.70)	<0.001
Race				
White	1			
Black	1.07 (0.85–1.35)	0.555		
Others	1.12 (0.81–1.56)	0.488		
Primary site				
Upper lobe	1		1	
Lower lobe	0.98 (0.85–1.12)	0.732	0.94 (0.82–1.08)	0.407
Middle lobe	0.94 (0.73–1.22)	0.652	1.06 (0.82–1.36)	0.675
Main bronchus	1.41 (1.08–1.83)	0.010	1.18 (0.90–1.54)	0.232
Overlapping lesion	4.33 (1.39–13.5)	0.012	5.32 (1.70–16.66)	0.004
Laterality				
Left	1			
Right	1.02 (0.90–1.16)	0.722		
Tumor size (cm)				
≤3	1		1	
3–4	1.26 (1.09–1.47)	0.002	1.09 (0.93–1.27)	0.288
4–5	1.21 (1.03–1.44)	0.024	1.08 (0.91–1.29)	0.365
Grade				
Moderate + well	1			
Poor	0.77 (0.47–1.27)	0.309		
Undifferentiated	0.97 (0.60–1.59)	0.913		
Unknown	1.13 (0.70–1.84)	0.609		
Surgery				
Non-surgery	1		1	
Lobectomy	0.36 (0.30–0.44)	<0.001	0.52 (0.39–0.68)	<0.001
Others	0.62 (0.51–0.75)	<0.001	0.62 (0.50–0.76)	<0.001
LNR (number)				
<4	1		1	
≥4	0.35 (0.28–0.43)	<0.001	0.55 (0.42–0.73)	<0.001
Chemotherapy				
No	1		1	
Yes	0.78 (0.69–0.89)	<0.001	0.64 (0.55–0.73)	<0.001
Radiotherapy				
No	1			
Yes	0.93 (0.82–1.05)	0.258		

LNR, lymph node removal; HR, hazard ratio; CI, confidence interval.

**Table 3:** Univariable and multivariate Cox proportional hazards regression analysis for lung cancer-specific survival.

Variables	Univariate cox regression		Multivariate cox regression	
	HR (95% CI)	P-value	HR (95% CI)	P-value
Sex				
Female	1			
Male	1.13 (0.98–1.29)	0.096		
Age				
<65 y	1		1	
≥65 y	1.40 (1.20–1.63)	<0.001	1.34 (1.15–1.56)	<0.001
Race				
White	1			
Black	1.09 (0.84–1.41)	0.517		
Others	1.14 (0.79–1.64)	0.479		
Primary site				
Upper lobe	1			
Lower lobe	1.04 (0.89–1.22)	0.601		
Middle lobe	0.91 (0.67–1.22)	0.517		
Main bronchus	1.43 (1.06–1.93)	0.018		
Overlapping lesion	3.42 (0.85–13.75)	0.083		
Laterality				
Left	1			
Right	1.03 (0.90–1.19)	0.656		
Tumor size (cm)				
≤3	1		1	
3–4	1.28 (1.08–1.52)	0.005	1.08 (0.91–1.29)	0.379
4–5	1.34 (1.11–1.61)	0.002	1.18 (0.98–1.43)	0.088
Grade				
Moderate + well	1			
Poor	0.96 (0.52–1.77)	0.885		
Undifferentiated	1.23 (0.67–2.26)	0.494		
Unknown	1.38 (0.76–2.52)	0.287		
Surgery				
None	1		1	
Lobectomy	0.33 (0.26–0.41)	<0.001	0.47 (0.34–0.66)	<0.001
Others	0.56 (0.45–0.70)	<0.001	0.56 (0.44–0.72)	<0.001
LNR (number)				
<4	1		1	
≥4	0.31 (0.24–0.40)	<0.001	0.53 (0.38–0.75)	<0.001
Chemotherapy				
No	1		1	
Yes	0.79 (0.68–0.91)	0.002	0.62 (0.53–0.73)	<0.001
Radiotherapy				
No	1			
Yes	0.88 (0.77–1.01)	0.070		

LNR, lymph node removal; HR, hazard ratio; CI, confidence interval.

### Developing the prognostic nomogram model for OS and LCSS

**Figure 2:**
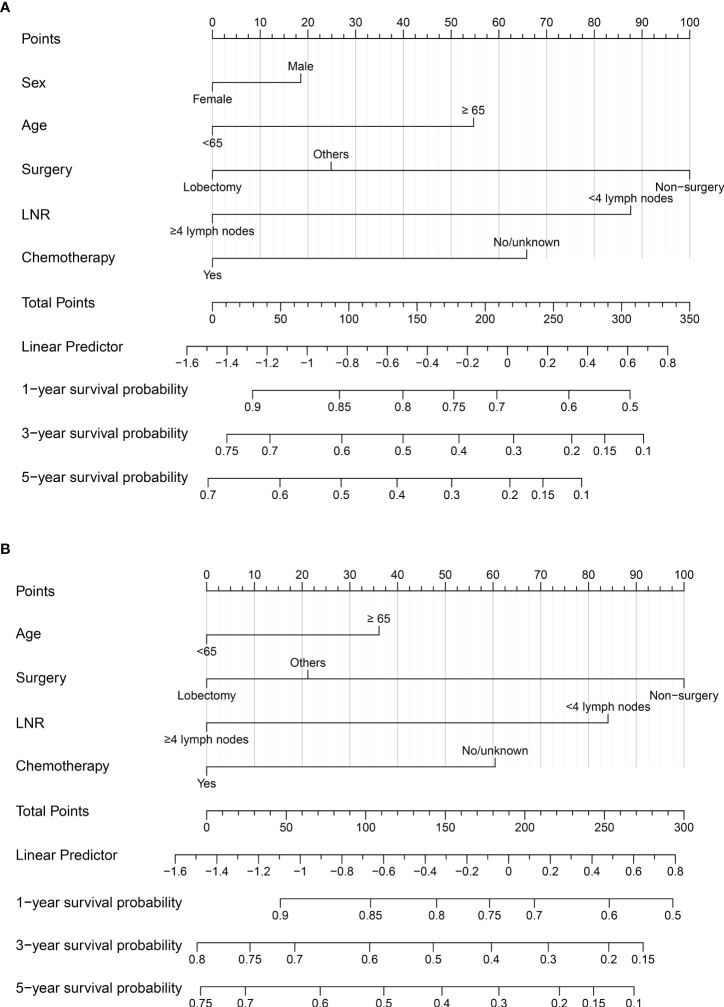
**(A)** Nomogram to predict 1-, 3-, and 5-year OS probability for early-stage T1-2N0M0 SCLC patients; **(B)** Nomogram to predict 1-, 3-, and 5-year LCSS probability for early-stage T1-2N0M0 SCLC patients. LNR, lymph nodes removal; SCLC, small cell lung cancer; OS, overall survival; LCSS, lung cancer-specific survival.

Significant variables of age, surgery, LNR, and chemotherapy were finally selected for the development of a nomogram model for 1-, 3-, and 5-year LCSS (**Figure 2B**). Besides, the variable of sex was also used to develop the nomogram model for 1-, 3-, and 5-year OS (**Figure 2A**). Each variable was assigned a point score ranging from 0 to 100. In the nomograms for OS and LCSS, the surgical procedure showed the largest contribution to prognosis, with a score of 100, followed by LNR. The factor of LNR showed a larger contribution in the nomogram model for OS than for LCSS, with a point of 87.5. Notably, the lobectomy demonstrated a great influence on survival prediction regardless of OS or LCSS. Each factor can be assigned a corresponding point by drawing a line straight upward to the “Point axis.” Individual risk scores were calculated by summing up the scores of each variable. The probabilities of survival at 1, 3, and 5 years were easily determined by locating their corresponding points on the survival scale.

### Calibration and validation of the nomogram

There were 344 events (deaths) in the validation cohort, of which 265 patients died of cancer. The mean follow-up duration was 30.34 months (median, 24 months; range, 1–83 months).

**Figure 3:**
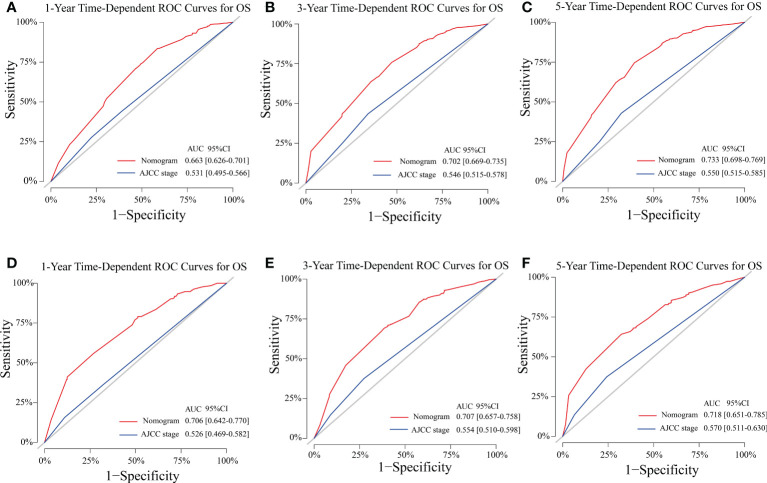
Model performance of the proposed nomogram. **(A–F)** Time-dependent ROC curves of the two prognostic models for predicting 1-, 3-, and 5-year OS. The AUCs of the two prognostic models at each time point of interest were presented in the training **(A–C)** and validation cohorts **(D–F)**. ROC, receiver operating characteristic; AUC, area under the curve; OS, overall survival; AJCC, American Joint Committee on Cancer; CI, confidence interval.

**Figure 4:**
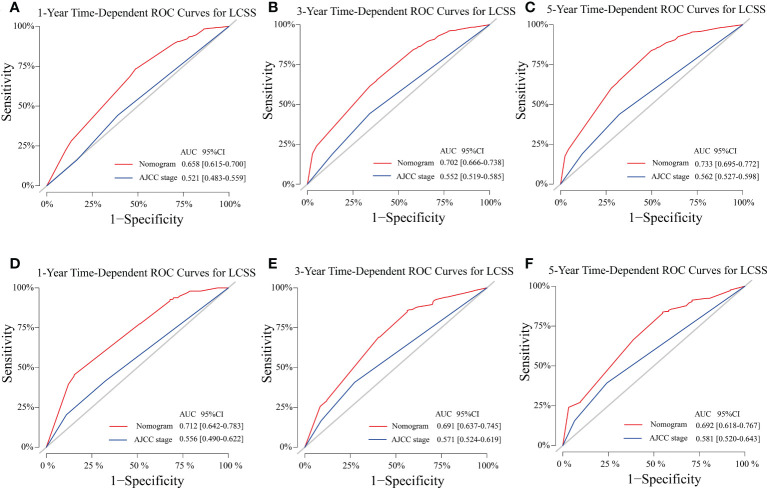
Model performance of the proposed nomogram. **(A–F)** Time-dependent ROC curves of the two prognostic models for predicting 1-, 3-, and 5-year LCSS. The AUCs of the two prognostic models at each time point of interest were presented in the training **(A–C)** and validation cohorts **(D–F)**. ROC, receiver operating characteristic; AUC, area under the curve; LCSS, lung cancer-specific survival; AJCC American Joint Committee on Cancer; CI confidence interval.

**Figure 5:**
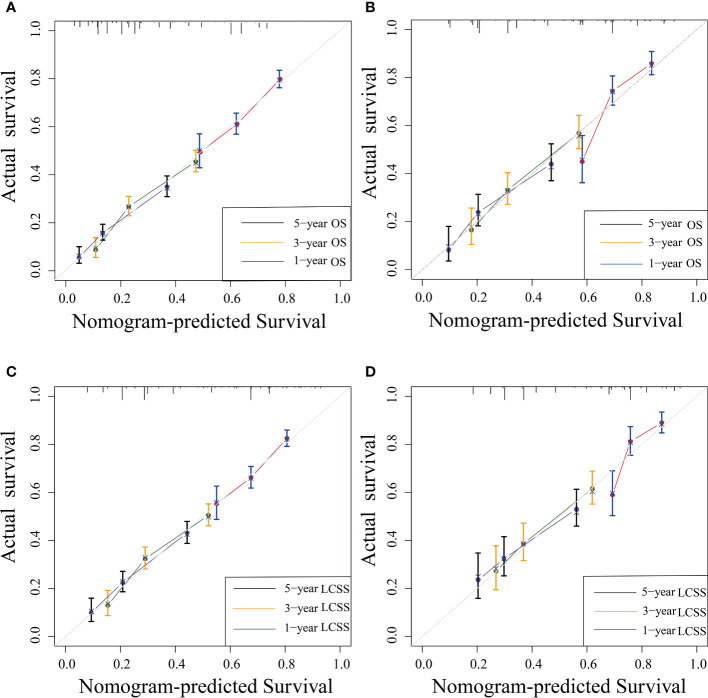
Nomogram calibration curves for nomogram-predicted survival (x-axis) and actual observed survival (y-axis). Calibration curves for OS **(A, B)** and LCSS **(C, D)** in the training **(A, C)** and validation cohort **(B, D)**; curves for 1-, 3-, and 5-year OS and LCSS were present as blue, yellow, and black lines, respectively. OS, overall survival; LCSS, lung cancer-specific survival.

**Figure 6:**
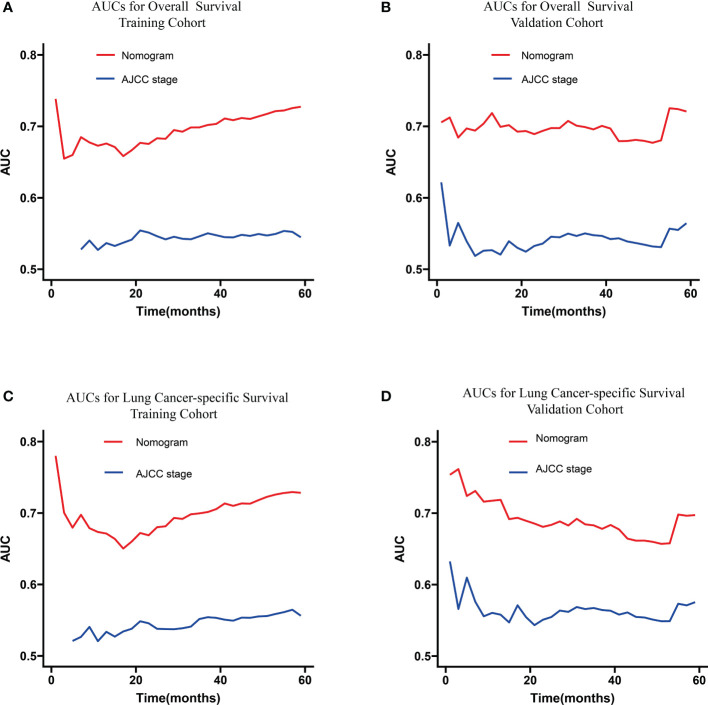
Continuous AUCs of the nomogram (red) and 8th edition AJCC TNM staging system (blue) in the training **(A, C)** and validation cohorts **(B, D)** throughout the period of 1–60 months. AUC, area under the curve; AJCC, American Joint Committee on Cancer.

**Figure 7:**
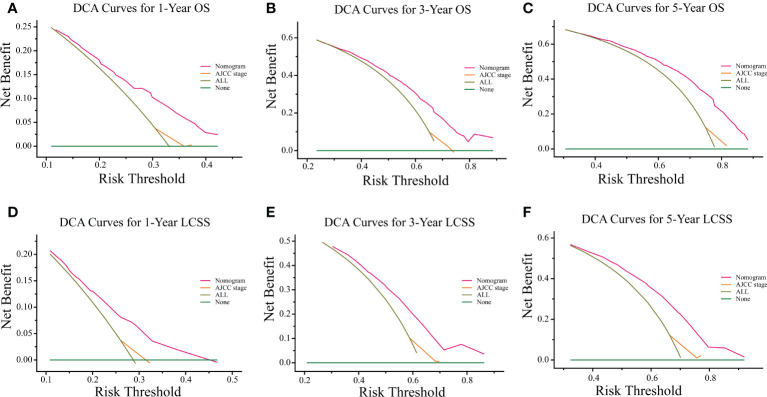
DCA curves of the proposed nomogram and 8th edition AJCC TNM staging system for 1-, 3-, and 5-year OS **(A–C)** and LCSS **(D–F)**. The x-axis represents the risk threshold, and the y-axis measures the net benefit. The green horizontal solid line along the x-axis assumes that overall death occurred in no patients, whereas the light green solid line assumes that all patients will have overall death at a specific threshold probability. The pink solid line represents the nomogram. The orange solid line represents the 8th edition AJCC TNM staging system. DCA, decision curve analysis; OS, overall survival; LCSS, lung cancer-specific survival; AJCC, American Joint Committee on Cancer.

The C-indexes of the training cohort were 0.663 (95% confidence interval [CI] 0.626–0.701), 0.702 (95% CI 0.669–0.735), 0.733 (95% CI 0.698–0.769) for 1-, 3-, and 5-year OS and 0.658 (95% CI 0.615–0.700), 0.702 (95% CI 0.666–0.738), 0.733 (95% CI 0.695–0.772) for 1-, 3-, and 5-year LCSS, respectively. In the validation cohort, they were 0.706 (95% CI 0.642–0.770), 0.707 (95% CI 0.657–0.758), 0.718 (95% CI 0.651–0.785) for 1-, 3-, and 5-year OS and 0.712 (95% CI 0.642–0.783), 0.691 (95% CI 0.637–0.745), 0.692 (95% CI 0.618–0.767) for 1-, 3-, and 5-year LCSS, respectively. The data indicated the brilliant discrimination ability of the nomogram (**Figures 3**, **4**). These calibration curves of the training cohort and validation cohort also presented acceptable consistency between the model prediction and the actual observation for 1-, 3-, and 5-year OS and LCSS (**Figure 5**). Concerning the prognostic ability of OS and LCSS, we conducted comparisons of the model performance between our nomograms and the 8th edition AJCC TNM staging system. The 1-, 3-, and 5-year time-dependent ROC curves of the two models are shown in **Figures 3**, **4**. All AUCs of the nomogram model were significantly higher than the 8th edition AJCC TNM staging system in the training cohort (**Figures 3A–C**, **4A–C**) and validation cohort (**Figures 3D–F**, **4D–F**), which verified the strong and robust prognostic power of our nomograms. Furthermore, we also compared the continuous trends of the prognostic performance of each model and found the AUCs of our nomogram models were significantly higher than that of the 8th edition AJCC TNM staging system throughout the calculation period (from months 1–60), whether in the training (**Figures 6A** **, C**) or validation cohorts (**Figures 6B** **, D**). After that, the DCA analysis suggested a significantly increased net benefit of the new nomogram over the 8th edition AJCC TNM staging system with wide and practical ranges of threshold probabilities regardless of the OS (**Figures 7A** **–C**) or LCSS (**Figures 7D** **–F**), which further verified the better individual prognostic performance of our nomograms in the clinical appliance.

### Performance of the new risk stratification model

**Figure 8:**
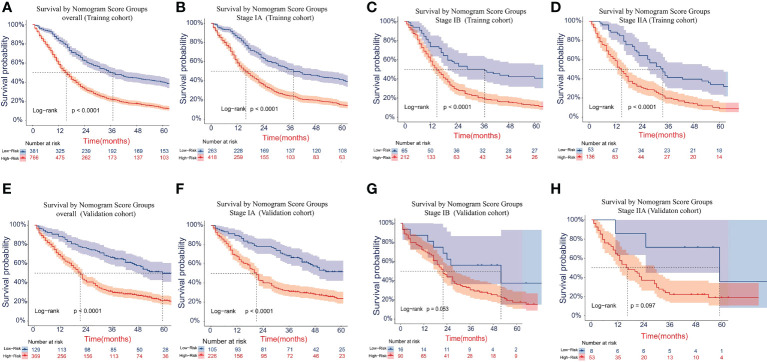
Kaplan–Meier survival curves for OS in the overall and stage-stratified patients in the training **(A–D)** and validation **(E–H)** cohorts to test the risk stratification system based on the training cohort. The red line represents the high-risk group, and the blue line represents the low-risk group. OS, overall survival.

**Figure 9:**
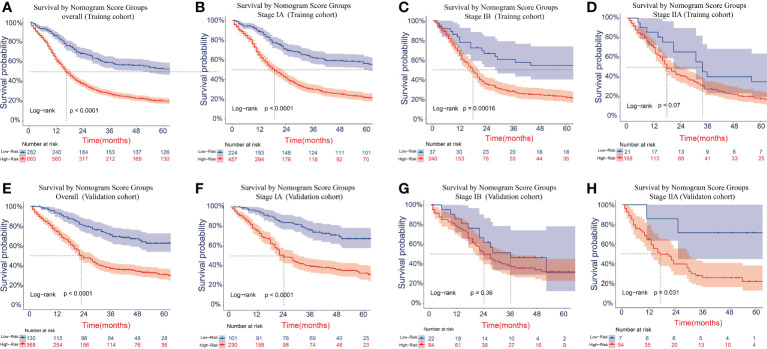
Kaplan–Meier survival curves for LCSS in the overall and stage-stratified patients in the training **(A–D)** and validation **(E–H)** cohorts to test the risk stratification system based on the training cohort. The red line represents the high-risk group, and the blue line represents the low-risk group. LCSS, lung cancer-specific survival.

The cutoff values for the high-risk group and low-risk group developed from X-tile software were 196.0 and 181.0 for OS and LCSS, respectively. All 1,147 patients in the training cohort were divided into the high-risk group (total points >196.0 for OS, >181.0 for LCSS) and the low-risk group (total points ≤196.0 for OS, ≤181.0 for LCSS) based on the cutoff value. The survival curves for OS and LCSS showed significant distinctions between the two different risk groups in the training cohort (P <0.0001, **Figures 8A**, **9A**). Significant differences in OS and LCSS were also observed between almost all subgroups when patients were stratified by AJCC stages (P <0.001, **Figures 8B–D**, **9B–D**). The same grouping method was then applied to the validation cohort, and significant distinctions in survival curves for OS and LCSS between the two different risk groups were also observed, even within certain AJCC staging categories (**Figures 8E–H**, **9E–H**).

### Development of web servers for the nomogram

For the convenient application of our nomogram, we created a user-friendly website. The website can calculate an individualized survival probability as long as you input certain clinical variables of a T1-2N0M0 SCLC patient and a certain prediction time (months). After that, it can also provide the corresponding survival plot for this case. The public online version of our nomogram is available at https://shanghaisuzhousclcnomogrampredictability.shinyapps.io/DynNomapp/ and https://shanghaisuzhousclcnomogrampredictability.shinyapps.io/DynNomappLCSS/. Clinicians can use the websites freely and do not need to input any passwords.

## Discussion

SCLC is well recognized as an easily aggressive tumor that will present hematogenous metastases and lymph node metastases at an early stage. So T1-2N0M0 SCLC is a relatively uncommon clinical scenario. Existing VALSG or TNM staging systems are not efficient in predicting the individual prognosis of early-stage T1-2N0M0 SCLC patients. Therefore, we constructed and validated novel nomogram prognostic models for OS and LCSS based on surgery and other clinicopathological variables to compensate for these limitations using a large population-based database of T1-2N0M0 SCLC patients. To our knowledge, this was the first comprehensive nomogram to provide a personalized predictive model for the OS and LCSS of early-stage T1-2N0M0 SCLC patients. Our new model demonstrated considerable discrimination ability and calibration accuracy both in the training and validation cohorts, which displayed good repeatability and reliability compared to the established model. After that, the nomograms showed a significant benefit in clinical application compared with the 8th TNM staging system through DCA analyses. Our risk stratification model depended on our nomograms and also could effectively stratify different risk patients by distinguishing OS and LCSS. So our models could help clinicians assess the survival of early-stage SCLC patients and better weigh the risks and benefits of more aggressive or more conservative anticancer therapies.

Several previous studies have published nomograms regarding survival prediction for SCLC patients. Xie and colleagues developed a nomogram using a cohort of 938 cases to predict OS for SCLC, incorporating peripheral blood markers (4). However, neither the study nor the independent validation assigned to the model applied the more accurate TNM staging system, nor did they assign an independent validation for the model. Due to the lack of early-stage T1-2N0M0 SCLC competing risk analyses in their model, we cannot compare the results between Xie’s model and our model in this study. In 2017, Wang et al. developed a prognostic nomogram for predicting the survival of SCLC patients using the National Cancer Database (NCDB) (20). This study applied the 8th TNM staging system and treatment patterns. However, the model incorporated the entire stages, which failed to provide accurate prediction for a special subset of patients. Besides, this study did not refer to the surgical procedure or the status of lymph node removal. While in 2021, Zeng et al. demonstrate a nomogram model for OS of resected limited-stage SCLC patients using the SEER database and an independent SCLC cohort at their single institution (21). Their predictive model could not only provide an accurate prediction for resected limited-stage SCLC but also provide the specific surgical procedure and lymph node status. But the model involves different stages, not merely the early-stage T1-2N0M0. Above all, prognostic models did not reveal predictors of death resulting from lung cancer-specific causes. Li and colleagues investigated the mortality of stage I SCLC patients in the presence of competing risks and conducted nomograms to predict probabilities of both lung cancer-specific death and death resulting from other causes (22). This study applied the 6th staging system and simply pointed out whether surgery was necessary or not without mentioning the concrete surgical procedure or lymph nodes removed, which means it might not be completely suitable for stage I SCLC patients. In contrast, our new model was constructed specially for early-stage T1-2N0M0 patients based on the characteristics of patients and tumor biology using a large population database and included common surgical procedures and the removal of lymph nodes. Besides, our new model was the first study to conduct the prediction of LCSS to provide the most beneficial treatment modalities and a more accurate probability of survival for this specific subset of patients. Notably, our new model received an ideal C-index by independent validation, so it has certain generalizability and can provide an accurate prediction.

Through univariate and multivariate analysis, sex, age, surgery, LNR, and chemotherapy were recognized as independent prognostic parameters of OS. Some of these factors have been studied in previous research on the survival of SCLC (23–27). After that, we also find that age, surgery, LND, and chemotherapy were associated with the LCSS by COX regression analyses. Sex did not affect lung cancer-specific mortality, which was consistent with the studies of Li et al. (22). But the male patients had worse OS than the female patients. Our study found the factor of age showed a larger contribution in the nomogram model for OS than for LCSS, which means that old patients may be more likely to die from other causes. Elder patients had worse survival than younger patients, which might be because of degenerative changes in various aspects of organ function and an increased prevalence of all types of comorbidities (28). So elder patients may require additional treatment and intensive follow-up. Our study showed that patients diagnosed with T1-2N0M0 SCLC without any lymph node metastasis should also should undergo surgical resection of lymph nodes. And the ones with more lymph node removal performed had better survival regardless of OS or LCSS, which suggested conducting lymphadenectomy for early-stage T1-2N0M0 SCLC patients. This result was similar to the results of Zeng et al. and Yang et al. (21, 29). Notably, the surgical procedure was a crucial independent predictor for OS and LCSS in our study, of which lobectomy posed the superior choice with better survival. There have been similar results in the previous study (7, 14, 30, 31). In our study, although the detailed location of the tumor was further refined based on lobes, the association between prognosis and the location of the tumor remained nonsignificant. In addition, we also find that there was no association between tumor size and prognosis in this subset of SCLC patients, regardless of the OS or LCSS.

However, several limitations existed in our study. First, certain biases may exist due to the inherent nature of this retrospective study. Second, certain shortcomings existed in using the SEER database, which lacked routinely available data including performance score, smoking status, pulmonary functions, body index, and comorbidities. Especially the variable of comorbidity will affect physicians in deciding the treatment strategies and evaluating the prognosis. After that, some available clinicopathological information about the patients is incomplete (e.g., the status of lymph node resection is unknown). Due to the dependence on the SEER database, we cannot conduct a more predictive survival analysis that includes the above-mentioned parameters. Moreover, several treatment-related variables were not included in our models, such as the sequence of chemotherapy and radiotherapy with surgery, the plans of chemotherapy, the number of cycles, the doses and methods of radiotherapy, and the further treatment after recurrence, so that our study cannot be able to evaluate the effects of treatment sequence, regimens, and courses on patients’ survival. We cannot externally validate the nomogram using data from our institution because of severe data loss. Hence, there should be a further multicenter prospective study that incorporates relatively complete clinicopathological variables and detailed information on treatment to create a more precise predictive model. But we considered that our model for OS and LCSS, conducted depending on other vital clinical factors that could be obtained in the SEER database with a larger sample, could provide some valuable implications in clinical practice for early-stage T1-2N0M0 SCLC patients.

In conclusion, our study found that the selection of surgical procedures was a crucial factor and that lymph node removal should be stressed even in node-negative SCLC patients because it was positively related to prognosis. Additionally, we developed and validated a prognostic nomogram for predicting 1-, 3-, and 5-year OS and LCSS in early-stage T1-2N0M0 SCLC patients with good discrimination and calibration, which has not been proposed in previous studies. Our model also showed certain reliability and could provide clinician suggestions to improve the prognosis, make treatment strategies, and design clinical trials. Besides, we implemented the nomogram in a user-friendly web server for clinicians and patients.

## Data availability statement

The raw data supporting the conclusions of this article will be made available by the authors, without undue reservation.

## Author contributions

All authors participated in manuscript writing and approved the final version of the manuscript. TG, SZ, LS, and LY conceived and designed the analysis. Collection and assembly of data were performed by TG, SZ, JQ, and XC. Analysis and interpretation of the data were supported by TG, SZ, LS, and JD. PZ, JZ, and GJ conducted a critical review of the manuscript, contributing important intellectual content. All authors contributed to the article and approved the submitted version.

## Funding

This work was supported by the Clinical Research Plan of SHDC (Grant No. SHDC2020CR2020B), the Funding of Shanghai Pulmonary Hospital (Grant No.FKCX1904, No.FKLY20004), and the Oasis Scholars Funding of Shihezi University.

## Conflict of interest

The authors declare that the research was conducted in the absence of any commercial or financial relationships that could be construed as a potential conflict of interest.

## Publisher’s note

All claims expressed in this article are solely those of the authors and do not necessarily represent those of their affiliated organizations, or those of the publisher, the editors and the reviewers. Any product that may be evaluated in this article, or claim that may be made by its manufacturer, is not guaranteed or endorsed by the publisher.
